# Capacity for instantaneous catabolism of preferred and non-preferred carbon sources in *Escherichia coli* and *Bacillus subtilis*

**DOI:** 10.1038/s41598-018-30266-3

**Published:** 2018-08-06

**Authors:** Marieke F. Buffing, Hannes Link, Dimitris Christodoulou, Uwe Sauer

**Affiliations:** 1 Institute of Molecular Systems Biology, ETH Zurich, Zurich, Switzerland; 2 Life Science Zurich PhD Program on Systems Biology, Zurich, Switzerland; 3 Max Planck Institute for Terrestrial Microbiology, Marburg, Germany

## Abstract

Making the right choice for nutrient consumption in an ever-changing environment is a key factor for evolutionary success of bacteria. Here we investigate the regulatory mechanisms that enable dynamic adaptation between non-preferred and preferred carbon sources for the model Gram-negative and -positive species *Escherichia coli* and *Bacillus subtilis*, respectively. We focus on the ability for instantaneous catabolism of a gluconeogenic carbon source upon growth on a glycolytic carbon source and vice versa. By following isotopic tracer dynamics on a 1–2 minute scale, we show that flux reversal from the preferred glucose to non-preferred pyruvate as the sole carbon source is primarily transcriptionally regulated. In the opposite direction, however, *E. coli* can reverse its flux instantaneously by means of allosteric regulation, whereas in *B. subtilis* this flux reversal is transcriptionally regulated. Upon removal of transcriptional regulation, *B. subtilis* assumes the ability of instantaneous glucose catabolism. Using an approach that combines quantitative metabolomics and kinetic modelling, we then identify the additionally necessary key metabolite-enzyme interactions that implement the instantaneous flux reversal in the transcriptionally deregulated *B. subtilis*, and validate the most relevant allosteric interactions.

## Introduction

Evolutionary adaptation enables bacterial growth and survival in a large range of habitats, as is evident from the ability to exploit diverse and frequently changing energy and nutrient sources^1^. Rapid proliferation depends on a microorganism’s ability to efficiently adapt its metabolism to nutritional changes, in particular those that affect the main carbon and energy source. Mechanisms of metabolic regulation range from modulation of enzyme activity through thermodynamics, allosteric regulation, or post translational modification to alteration of enzyme availability through transcriptional regulation and protein degradation. The specific implementation of the regulatory mechanisms in a given microorganism can vary and is influenced by the environmental challenges of its natural environment^2–7^.

The classic example of metabolic adaptation to an alternative carbon source are diauxic shifts of *Escherichia coli* from a preferred carbon source such as the glycolytic glucose to typically less preferred gluconeogenic carbon sources such as acetate, pyruvate, or succinate^8^. These carbon sources are used in a hierarchical order of preference that is transcriptionally regulated by carbon catabolite repression, a process that prevents expression of enzymes for catabolism of less preferred carbon sources when the preferred substrate is present^9–11^. Given this hierarchy, it is generally assumed that microbes are always ready to instantaneously consume their preferred carbon sources, glucose for many bacteria^9,12^. For *E. coli* it has indeed been shown that glucose can instantaneously be taken up and metabolized during growth on the non-preferred carbon source pyruvate^13^. For *Bacillus subtilis*, however, glucose uptake was shown to be delayed when added during growth on malate^14^, a second preferred substrate of the organism^15,16^.

Here, we investigate the capacity of *E. coli* and *B. subtilis* for instantaneous consumption and metabolism of preferred and non-preferred glycolytic and gluconeogenic carbon sources. For this purpose we performed isotopic tracer-based carbon source shift-experiments at a time-scale below two minutes. By quantifying ^13^C-label integration (or dilution), we distinguish uptake and further metabolism of the alternative carbon source and provide a direct readout for glycolytic flux reversal. Both bacteria exhibit a similar delay for metabolism of the non-preferred carbon source pyruvate. They differ, however, fundamentally in their ability to instantaneously utilize glucose. We demonstrate that instantaneous glucose uptake and catabolism during growth on pyruvate is transcriptionally prevented in *B. subtilis*. Upon removal of this transcriptional regulation, we used kinetic modelling to demonstrate that rapid glycolytic flux reversal requires additional allosteric regulation and verify the predictions through *in vitro* experiments.

## Material and Methods

### Strains and media

The wild-type strains were *E. coli* BW 25113 and *B. subtilis* 168 trp^+^, and the mutant strains *B. subtilis glcT* and *ΔcggR glcT(Con)* were previously described^17^. For cultivation, frozen glycerol stocks were used to inoculate 5 mL Luria-Bertani (LB) medium. Glycerol stocks of *B. subtilis* mutants were first plated on LB-plates with antibiotics (kanamycin (50 μg mL^−1^) for *B. subtilis glcT(Con)* and kanamycin (50 μg mL^−1^) + phleomycin (10 μg mL^−1^) for *B. subtilis ΔcggR glcT(Con)*. Single colonies were then used to inoculate LB medium. LB pre-cultures were grown for 5–6 hours at 37 °C. The LB pre-cultures were used to inoculate M9 pre-culture medium, which was grown overnight at 37 °C. An aliquot of the M9 pre-cultures was used to inoculate 30 mL M9 medium with an initial optical density at 600 nm (OD_600_) of 0.05.

The M9 minimal medium contained per litre of deionized water 8.5 g Na_2_HPO_4_ × 2H_2_O, 3 g KH_2_PO_4_, 1 g NH_4_Cl, and 0.5 g NaCl, pH was adjusted to pH 7.0 using 4 M NaOH prior to autoclaving. The following components were filter sterilized separately and then added (per litre of final medium): 1 mL 0.1 M CaCl_2_ × 2H_2_O, 1 mL 1 M MgSO_4_ × 7H_2_O, 1 mL 100 mM FeCl_3_ × 6H_2_O, and 10 mL trace salt solution. The trace salt solution contained per litre 170 mg ZnCl_2_, 100 mg MnCl_2_ × 4H_2_O, 60 mg CoCl_2_ × 6H_2_O, 60 mg Na_2_MoO_4_ × 2H_2_O, and 43 mg CuCl_2_ × 2H_2_O. Glucose, malate, and pyruvate were filter sterilized and pH neutralized before addition to the medium. The final concentration of the carbon sources were 3 g L^−1^ for glucose, 5 g L^−1^ for malate, 5 g L^−1^ for pyruvate, and for malate/glucose 4 g L^−1^ malate and 2 g L^−1^ glucose respectively. For the ^13^C-trace experiments glucose was replaced by uniformly labelled [U-^13^C] glucose (^13^C ≥ 99%, Cambridge Isotope Laboratories) in the same concentrations.

### Carbon source switches

Shake flask cultures were grown exponentially to an OD_600_ of 0.4–0.6 for removal of 2 mL aliquots by vacuum filtration on a 0.45-μm pore size Hydrophilic PVDF filter (Millipore). On the filter, cells were first perfused for 10 s with the M9 medium used for the shake flask cultures, after which immediate substrate switches were achieved by changing the perfusion solution to M9 medium with the alternative carbon source^13^. Perfusion solutions were kept at 37 °C.

### Metabolite measurements

Filters with cultured cells were immediately transferred for extraction into 2:2:1 acetonitrile:methanol:water solution at −20 °C. Metabolites were extracted for at least 2 hours and stored at −80 °C until further processing. For absolute metabolite quantification, 100 μL ^13^C-internal standard (a metabolite extract of *E. coli* grown on [U-^13^C]glucose) was added to the extraction solution^17^. Extracts were centrifuged at 14 000 g at 4 °C for 20 min to remove cell debris. Supernatants were dried at 0.12 mbar to complete dryness in a SpeedVac composed of an Alpha 2–4 LD plus cooling trap, a RVC 2–33 rotational vacuum concentrator and a RC-5 vacuum chemical hybrid pump (Christ, Osterode am Harz, Germany). Dry metabolite extracts were stored at −80 °C until further analysis.

Dried metabolite samples were resuspended in 100 μL deionized water, of which 10 μL was injected into a Waters Acquity UPLC with a Waters T3 column (150 mm × 2.1 mm × 1.8 mm; Waters Corporation, Milford, MA) coupled to a Thermo TSQ Quantum Ultra triple quadrupole instrument (Thermo Fisher Scientific, Waltham, MA) with electrospray ionization. Peak integration was performed with in-house software. ^13^C-traces and metabolite concentrations were determined as previously described^17,18^.

### Kinetic modelling

The kinetic glycolysis/gluconeogenesis model of *B. subtilis* consisted of 8 ordinary differential equations. MATLAB 2013a was used for all simulations and calculations. The transport reactions and irreversible reactions were described by Michaelis-Menten kinetics, whereas reversible reactions were modelled with mass action kinetics.

The base model had 16 free parameters, i.e. K_M_-values, V_max_-values, and steady state concentrations of compounds that were not measured experimentally (Supplement). Models representing pairwise interactions had an additional 2 parameters that account for the two allosteric interactions tested with a sampled strength. Allosteric regulation was implemented as a power law term with the exponent termed alpha for all possible pairwise combinations of allosteric interactions between all metabolites and enzymes in the model (Supplement). For parameter estimation, we sampled 0.1–10 fold around the literature K_M_-values, and 0.5–2 fold around the K_M_-values determined in this study for *B. subtilis* PfkA and PycA (Table 1). Steady state concentrations of pyruvate and oxaloacetate were sampled from 0–10 mM due to lack of experimental data. The interaction parameter alpha was sampled in the fold range from −4 for inhibition to 4 for activation. We allowed maximum pyruvate uptake rate to fluctuate around 1.15 mM sec^−1^ (in a range of 0.02–1.18 mM sec^−1^) and the maximum glucose uptake rate was 1.06 mM sec^−1^^19^ (Table S1). Futile cycling between Pfk/Fbp and GapA/GapB varied in the range 50–100%. The free parameters of the base model were sampled 10’000 times, and of the pairwise models 1000 times. To rank the models, we evaluated the root mean square error between simulations and experimental data, penalizing for the extra interactions (the parameter alpha) using the Akaike Information Criterion (AIC). This, combined with the frequency a pairwise model was outperforming the base model, formed the total score of a model. A detailed description of the model and the scoring can be found in the supplementary information.

**Table 1:** Reactions and kinetic parameters of *B. subtilis* used for modelling.

Enzyme	Enzyme (full name)	EC	Reaction	K_M_ (mM)	Reference
**Transport**					
PTSg	phosphotransferase system	2.7.1.9	glucose + PEP → G6P + pyruvate	20	^43^
**Irreversible reaction**					
G6PDH	glucose 6-phosphate dehydrogenase	1.1.1.49	G6P + NADP ↔ 6PG + NADPH + H	0.075	^44^
Pfk	6-phosphofructokinase	2.7.1.11	F6P + ATP ↔ FBP + ADP + H	0.12	this study
Fbp	fructose-1,6-biphosphatase	3.1.3.11	FBP + H_2_O ↔ F6P + P_i_	0.1	^35^
GapA	glyceraldehyde-3-phosphate dehydrogenase	1.2.1.12	GAP + NAD + P_i_ ↔ BPG + NADH + H	0.1	^21^
GapB	glyceraldehyde-3-phosphate dehydrogenase	1.2.1.13	BPG + NADPH + H ↔ GAP + NADP + P_i_	0.86	^21^
Pyk	pyruvate kinase	2.7.1.40	PEP + ADP + H ↔ pyruvate + ATP	0.25	^45^
PycA	pyruvate carboxylase	6.4.1.1	pyruvate + ATP + CO_2_ ↔ OAA + ADP + P_i_ + H	0.10	this study
Pdh	pyruvate dehydrogenase	1.2.4.1	pyruvate + NAD ↔ acetyl-CoA + NADH + CO_2_	0.4	^46^
PckA	phosphoenolpyruvate carboxykinase	4.1.1.49	OAA + ATP ↔ PEP + ADP + CO_2_	0.025	^22^
**Reversible reaction**					
Pgi	glucose 6-phosphate isomerase	5.3.1.9	G6P ↔ F6P	—	
Fba	fructose-1,6-biphosphate aldolase	4.1.2.13	FBP ↔ DHAP + GAP	—	
Tpi	triose phosphate isomerase	5.3.1.1	DHAP ↔ GAP	—	
Fba + Tpi ( = Ald)			FBP ↔ 2 DHAP	—	
Pgk	phosphoglycerate kinase	2.7.2.3	BPG + ADP ↔ 3PG + ATP	—	
Pgm	phosphoglycerate mutase	5.4.2.12	3PG ↔ 2PG	—	
Eno	enolase	4.2.1.11	2PG ↔ PEP + H_2_O	—	
Pgk + Pgm + Eno ( = Eno)			xPG ↔ PEP	—	

### Enzyme purification

The *B. subtilis* phosphofructokinase-encoding *pfkA* and pyruvate carboxylase-encoding *pycA* genes were amplified by PCR with Phusion high-fidelity DNA polymerase (BioLabs) using *B. subtilis* 168 trp^+^ genomic DNA as template. Primers of both genes had restriction sites for XhoI on the forward primer and XbaI on the reverse primer, as well as a His-tag on the 5′ end. The primers used were Fw_pfkA (GCC TCG AGA TGC ATC ATC ATC ATC ATC ATG GTG GTG GTA AAC GAA TAG GGG TAT TAA CGA GC) and Rv_pfkA (GCT CTA GAT TAG ATA GAC AGT TCT TTT GAA AGC TGA TA) for *pfkA* and Fw_pycA (GCC TCG AGA TGC ATC ATC ATC ATC ATC ATG GTG GTG GTT CTC AGC AAT CGA TAC AAA AAG TAT TAG) and Rv_pycA (GCT CTA GAT TAT GCT TTT TCA ATT TCA AGG AG) for *pycA*. After amplification and restriction, the product was ligated into pTrc99KK, an IPTG-inducible plasmid with ampicillin as a resistance marker^13^. Subsequently, Mix&Go competent *E. coli* cells (Zymo Research) were transformed with the overexpression plasmid and grown on plates with ampicillin (100 μg mL^−1^) (37 °C, O/N). Through restriction of the plasmid with XhoI and XbaI and sequencing, a successful clone was selected and the isolated plasmid was used to transform *E. coli* BW25113 by electroporation.

For purification of the *B. subtilis* enzymes PfkA and PycA, *E. coli* overexpression strains were grown overnight at 37 °C in LB with 100 μg mL^−1^ ampicillin and 200 μM IPTG. The cells were harvested by centrifugation (5 000 g, 4 °C, 20 min) and the pellet was washed with 10 mL 0.9% (w/v) NaCl with 10 mM MgSO_4_. The cells were lysed by French press in 4 mL lysis buffer (pH 7.4) containing 20 mM NaPO_4_, 0.5 M NaCl, 20 mM imidazole, 5 mM MgCl_2_, 2 mM DTT, and 4 mM PMSF. After centrifugation (14 000 g, 4 °C, 30 min), the proteins were His-tag purified with Ni-NTA affinity columns with 40 mg column^−1^ DBC (GE Healthcare), and the elution buffer was replaced by the respective assay buffer using filter columns with 10 kDa MWCO (Millipore). The concentrations of the purified enzymes were quantified based on bovine serum albumin (BSA) dilution series and the proteins were stored for maximum three days at 4 °C.

### *In vitro* enzyme assays

All substrate and co-factor solutions were prepared freshly. To determine the K_M_ value of *B. subtilis* PfkA, we used a coupled spectrophotometric assay^13^. The assay contained 2 µg mL^−1^ phosphofructokinase, 1.3 U mL^−1^ aldolase, 16.3 U mL^−1^ triosephosphate isomerase, 1.6 U mL^−1^ α- glycerophosphate dehydrogenase, 100 mM Tris-HCl (pH 8) 10 mM MgCl_2_, 0.2 mM NADH, 4 mM ATP, and varying concentrations of fructose 6-phosphate (0.01–2.5 mM) in a final volume of 200 µL at room temperature. For PycA we used a coupled spectrophotometric assay^20^ that contained 1 U mL^−1^ malate dehydrogenase, 135 mM TEA (pH 8), 7 mM MgSO_4_, 0.23 mM NADH, 1 mM ATP, 0.05 mM acetyl-coA, 15 mM KHCO_3_, and varying concentrations of pyruvate (0.04–5.0 mM) in a final volume of 200 µL at room temperature.

Reactions were initiated by adding the enzyme of interest and NADH consumption was monitored over time by recording the absorbance at 340 nm. K_M_ and K_i_ values were estimated by fitting the measured data in MATLAB 2015b. For the verification of allosteric regulators, we added the potential effector in varying concentrations before starting the reaction by adding enzyme.

## Results

### Inability for instantaneous gluconeogenesis of a non-preferred carbon source in *E. coli* and B. subtilis

**Figure 1:**
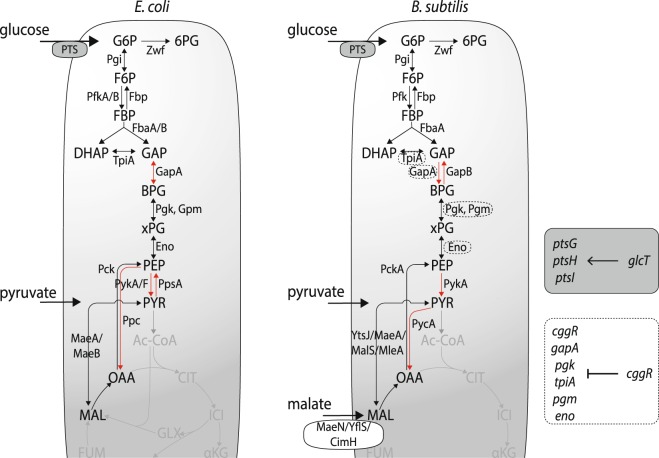
Glycolysis in *E. coli* and B. subtilis. Differences between the two organisms are highlighted in red. Metabolites: G6P = glucose 6-phosphate, F6P = fructose 6-phosphate, FBP = fructose 1,6-biphosphate, DHAP = dihydroxyacetone phosphate, GAP = glyceraldehyde 3-phosphate, BPG = 1,3-biphosphoglycerate, xPG = 2/3-phosphoglycerate, PEP = phosphoenol pyruvate, PYR = pyruvate, OAA = oxaloacetate, MAL = malic acid. Enzymes: Zwf = G6P dehydrogenase, Pgi = G6P isomerase, Pfk = 6-phosphofructokinase, Fbp = fructose-1,6-biphosphatase, Fba = FBP aldolase, Tpi = triose phosphate isomerase, Gap = GAP dehydrogenase, Pgk = phosphoglycerate kinase, Pgm = phosphoglycerate mutase, Eno = enolase, Pyk = pyruvate kinase, Pck = PEP carboxykinase, YtsJ = NADP-dependent malic enzyme, Mae = NAD-dependent malic enzyme, MalS = NAD-dependent malic enzyme, MleA = NAD-dependent malic enzyme, Pps = PEP synthetase, Pyc = PYR carboxylase, Ppc = PEP carboxylase. Transport: PTS = phosphotransferase system, ptsG = phosphotransferase system (PTS) glucose-specific enzyme IICBA component, ptsH = polypeptide: histidine-containing phosphocarrier protein of the phosphotransferase system (PTS) (HPr protein), ptsI = phosphotransferase system (PTS) enzyme I. Regulators: CggR = negative regulator of eno-operon, glcT = transcriptional antiterminator (BgIG family)

Glucose is the preferred carbon source of both *E. coli* and *B. subtilis* and is primarily catabolized via the glycolytic pathway^6,19^. Growth on typically non-preferred organic acids such as pyruvate, requires reversal of the glycolytic flux into the gluconeogenic direction to provide hexose and pentose phosphates as building blocks for biomass. Glycolysis and gluconeogenesis are similar in both species, but differ at three points (Fig. 1): (1) *B. subtilis* has two physiologically distinct glyceraldehyde-3-phosphate dehydrogenases with different cofactor specificities for growth on glycolytic or gluconeogenic carbon sources, whereas *E. coli* has only a single enzyme that operates in both flux directions;^21^ (2) *B. subtilis* lacks the gluconeogenic enzyme phosphoenolpyruvate (PEP) synthetase;^22^ and (3) the main anaplerotic reaction to replenish carbon in the tricarboxylic acid (TCA) cycle is pyruvate carboxylase in *B. subtilis* and PEP carboxylase in *E. coli*^23^. Since glucose represses the use of alternative carbon sources through carbon catabolite repression in both organisms^9^, one would expect a delay in reaching a growth-supporting change in gluconeogenic flux when replacing glucose with a non-preferred carbon source.

**Figure 2:**
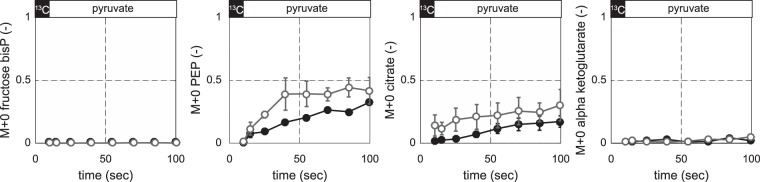
Isotope labelling dynamics during carbon source shifts from the preferred [U-^13^C]glucose to the non-preferred pyruvate. Labelling was initiated at time zero through feeding [U-^13^C]glucose and M + 0 denotes the unlabelled fraction of each metabolite. *E. coli* (grey, open dots), *B. subtilis* (black, filled dots).

To test whether the delay in utilization of a non-preferred carbon source takes place at the level of uptake or in the further metabolic conversion, we grew both species to a fully metabolic and isotopic steady state on uniformly labelled [U-^13^C]glucose and exposed them for 90 seconds to a medium containing unlabelled pyruvate as the single carbon source. Pyruvate is a representative gluconeogenic carbon source and allows for a direct entry at the start of gluconeogenesis. Since metabolism of pyruvate dilutes the ^13^C-label, increasing unlabelled ^12^C-fractions (F_M+0_) constitute a direct readout of flux reversal from the glycolytic to the gluconeogenic direction. Also, the closer an intermediate is to the entry point of the carbon source, the faster the F_M+0_ will increase. The label dilution in PEP, which is close to the pyruvate entry, shows that pyruvate is taken up in both organisms (Fig. 2). In *E. coli*, the F_M+0_ of PEP reaches 0.4 within 40 seconds and then plateaus, demonstrating pyruvate uptake and an initial flux in lower gluconeogenesis that stalls. In *B. subtilis*, the dilution dynamics are slower but reach a similar level after 90 seconds. Since fructose-1,6-bisphosphate (FBP) remained entirely labelled, complete gluconeogenesis cannot be established in either species within this short time frame. Label-dilution is also slow in the direction of the TCA cycle; i.e. citrate reaches F_M+0_ 0.2 after 90 seconds and alpha-ketoglutarate only reaches 0.1. Thus, both pyruvate uptake and its metabolism appear to limit immediate use of and growth on pyruvate in both species.

### B. subtilis cannot metabolize malate instantaneously into the gluconeogenic direction to support growth

**Figure 3:**
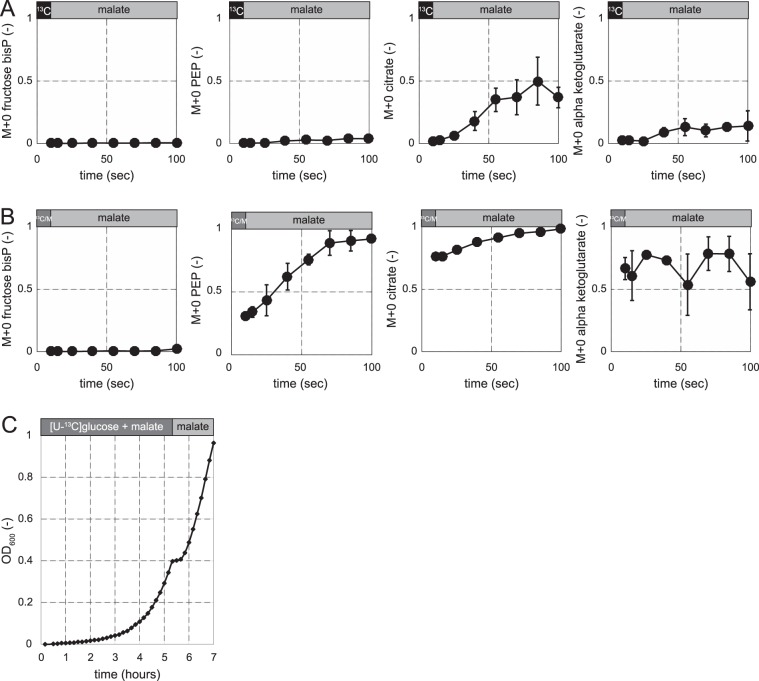
Isotope labelling dynamics during a shift from a preferred glycolytic to a preferred gluconeogenic carbon source in *B. subtilis*. (**A**) [U-^13^C]glucose was replaced by [^12^C]malate. (**B**) [U-^13^C]glucose + malate was replaced by [^12^C]malate. (**C**) Growth curve of *B. subtilis* during a diauxic shift when glucose depletes from a culture growing on glucose + malate. M + 0 denotes the unlabelled fraction of each metabolite.

For *B. subtilis*, glucose and the gluconeogenic malate are both preferred carbon sources that repress the use of alternative carbon sources^15,16,24^. Malate was also shown to be co-metabolized when added to cultures growing exponentially on glucose, showing that malate is not subject to carbon catabolite repression by glucose^14^. The capacity to immediately co-metabolize malate with glucose, however, does not necessarily imply that *B. subtilis* can use malate instantaneously as a single carbon source. Hence we investigated the capacity for instantaneous malate metabolism by following the label dilution of [U-^13^C]glucose-grown cultures that were switched to unlabelled malate as the single carbon source (Fig. 3A). Label dilution from the unlabelled malate propagated within 90 seconds to citrate (F_M+0_ 0.45), but barely to alpha-ketoglutarate (F_M+0_ 0.1) presumably due to repression of aconitase by CcpC in the presence of glucose^25^. Moreover, there was no apparent metabolism of malate into gluconeogenesis as PEP and FBP both remained completely labelled, presumably due to CcpN repression of PEP carboxykinase (PckA) and the gluconeogenic NADPH-dependent glyceraldehyde-3-phosphate dehydrogenase (GapB)^19,26,27^.

To circumvent CcpN repression and thereby enable flux reversal of lower glycolysis, we grew *B. subtilis* on [U-^13^C]glucose together with unlabelled malate. These co-metabolizing cultures were switched to unlabelled malate as the single carbon source and we followed label dilution (Fig. 3B). Without an apparent delay, malate was taken up and catabolized in the TCA cycle as evidenced by the label dilution of the citrate pool that reached isotopic steady state within 90 seconds, but further label dilution to alpha-ketoglutarate did not occur. Malate was also instantaneously metabolized into the gluconeogenic direction as indicated by the label dilution in PEP and 2/3-phosphoglycerate. Yet FBP remained completely labelled, indicating that hexoses cannot be immediately produced from malate after the shift and thus cell growth is not possible. This was further confirmed by an apparent lag phase of about half an hour when glucose was depleted from a culture growing on glucose and malate (Fig. 3C). The previously described immediate malate uptake^14^ combined with our results shows that instantaneous growth after a switch to malate as a single carbon source is limited by flux reversal of glycolysis and not by malate uptake. Overall, the results demonstrate that immediate and rapid uptake and catabolism of malate is possible through parts of central carbon metabolism where the flux direction does not need to revert. However, the inability to rapidly reverse flux from glycolysis to gluconeogenesis prevents *B. subtilis* from instantaneous growth on malate and causes a lag phase. Hence, the inability for a rapid flux reversal from glycolysis to gluconeogenesis is independent of the carbon source preference.

### Glucose uptake and metabolism is instantaneous in *E. coli* and enzyme-limited in B. subtilis

**Figure 4:**
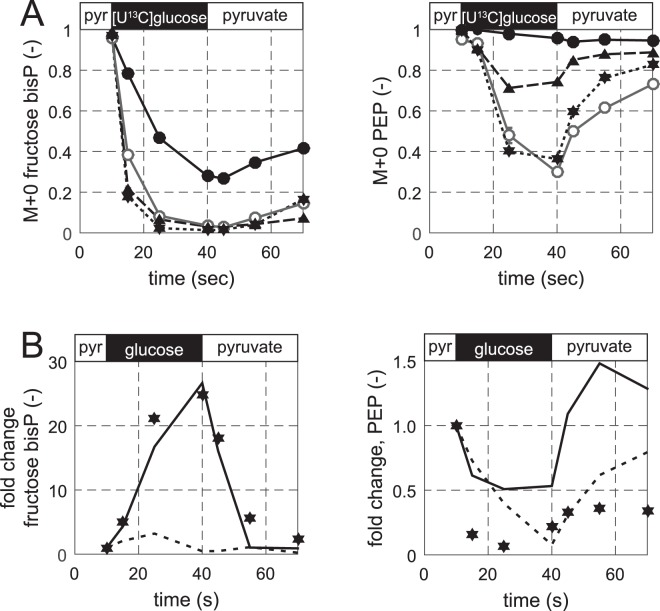
Metabolite and isotope dynamics during a shift from a non-preferred to a preferred carbon source and back. [^12^C]pyruvate was replaced by [U-^13^C]glucose and the culture was shifted back to [^12^C]pyruvate. (**A**) Isotope labelling dynamics in *E. coli* (grey solid line with open circles), *B. subtilis* 168 trp + wild type (black solid line with filled circles), *B. subtilis* constitutive glcT (black dashed line with triangles), and *B. subtilis* constitutive glcT and ΔcggR (black dotted line with stars). (**B**) Metabolite concentration changes in the transcriptionally deregulated strain *B. subtilis* constitutive glcT and ΔcggR. Experimental data are denoted by filled stars. Kinetic model predictions by the best base model and the model with the allosteric interactions PEP(-)Pfk and PEP(+)Fbp are denoted by the dashed and solid lines, respectively.

In the previous section, we demonstrated that glucose-grown *E. coli* and *B. subtilis* cultures retain a basal capacity for instantaneous catabolism of organic acids but cannot immediately and completely revert their glycolytic flux. It is a generally held belief that this catabolic capacity is maintained for the preferred carbon source, at least for glycolytic ones, during growth on gluconeogenic organic acids^28,29^. To test this presumption, we grew *E. coli* and *B. subtilis* exponentially on unlabelled pyruvate and exposed them for 30 seconds to a medium with [U-^13^C]glucose as the sole carbon source before switching back to pyruvate. Hence increasing ^13^C-labelled fractions in intracellular metabolite pools allow to conclude on uptake and metabolism of ^13^C-glucose. Consistent with existing data for *E. coli*^13^, we demonstrate instantaneous glucose metabolism as shown by the rapid decrease of the unlabelled fraction of FBP (F_M+0_ 0.03 within 30 seconds) and the further label propagation through glycolysis to PEP (F_M+0_ 0.30 within 30 seconds) (Fig. 4A). This rapid incorporation of glucose label demonstrates availability of the necessary enzymes and suggests that allosteric regulation, i.e. protein-metabolite interaction and/or post-translational modification, is sufficient to enable instantaneous flux reversal from gluconeogenesis to glycolysis in *E. coli*.

*B. subtilis*, however, exhibited a very different response with much slower label integration into FBP and almost no glycolytic flux to PEP (F_M+0_ 0.96 after 30 seconds) (Fig. 4A). Given the investigated time frame of below one minute, it is justified to assume that transcript levels remain unaltered at the level present during growth on the pre-shift medium, in this case with pyruvate as the single carbon source^19,30^. To test whether transcriptional regulation prevented instantaneous glucose catabolism, we used *B. subtilis* mutants that constitutively express the phosphotransferase system (PTS) and the *eno*-operon that had previously been identified as bottlenecks for co-metabolism of glucose with malate^14^. Specifically, we used a strain that constitutively expresses the transcription factor GlcT, which, in turn, leads to constant expression of the PTS operon^31^, and a strain deleted for the CggR repressor of the *eno*-operon (*cggR* – *gapA* – *pykA* – *tpiA* – *pgm* – *eno*)^32^. Constitutive expression of the PTS in the *B. subtilis glcT(Con)* mutant enabled instantaneous metabolism of glucose to FBP (F_M+0_ 0.03 within 30 seconds) and, to some extent, reversal of the gluconeogenic flux to PEP (F_M+0_ 0.74 within 30 seconds) (Fig. 4A). Additionally relieving repression of the *eno*-operon in the Δ*cggR glcT(Con)* mutant enabled label integration into PEP with similar dynamic as in *E. coli* (F_M+0_ 0.36 within 30 seconds). Thus, transcriptional regulation by GlcT and CggR prevents instantaneous glucose catabolism in *B. subtilis*.

### Identification of allosteric regulation required for glycolytic flux reversal in B. subtilis

In contrast to *E. coli*, *B. subtilis* transcriptionally represses both gluconeogenesis and glucose catabolism when grown on organic acids or glucose, respectively. A remaining question is whether or not this transcriptional regulation is the only mechanism to control flux reversal from gluconeogenesis to glycolysis. To test the hypothesis of additional regulation on the enzyme-level, we determined intracellular metabolite dynamics during the 30-second transition experiment from pyruvate to glucose, and back to pyruvate, in the transcriptionally deregulated *B. subtilis ΔcggR glcT(Con)* mutant. If constant expression of the PTS and eno-operon are sufficient, an ordinary differential equation (ODE) model of the relevant enzymes should be able to describe the measured metabolite dynamics without considering allosteric regulation. For this purpose, we constructed a kinetic base model of glycolysis/gluconeogenesis that consists of 8 ODEs that describe the dynamic changes of eight intracellular metabolites; i.e. glucose 6-phosphate, fructose 6-phospate, FBP, dihydroxyacetone phosphate, 2/3-phosphoglycerate, PEP, pyruvate, and oxaloacetate. Since pyruvate and oxaloacetate could not be measured, their initial intracellular concentrations were sampled within a range from 0–10 mM. We assumed mass action kinetics for the 3 reversible reactions and Michaelis-Menten kinetics for the 10 irreversible reactions. To compensate for the differences in laboratory conditions, we randomly sampled the Michaelis-Menten constants (K_M_) one order of magnitude below and above the literature values (Table 1). Since the K_M_ values for PycA and PfkA were unknown, we determined them through *in vitro* enzyme assays with isolated enzymes as K_M,pycA_ = 0.10 mM pyruvate and K_M,pfkA_ = 0.12 mM fructose 6-phosphate and we sampled 0.5–2 times around those values to compensate for the difference of *in vitro* and *in vivo* conditions^33^. From the flux distributions during steady state growth on glucose (Table S1), we calculated the maximum reaction rates for each reaction (V_max_).

Subsequently, we used the base model to simulate the dynamic intracellular metabolite concentrations with the free parameters sampled (Supplement). The quality of the simulated models was scored based on the sum of squared errors between the simulated and experimental data^34^. There was no set of parameters that allowed the base model to capture the measured metabolite dynamics, suggesting the relevance of an additional regulation mechanism (Fig. 4B). To test for such putative allosteric regulation, we constructed a set of structurally different models in which the base model was extended with up to two additional parameters that describe either activation or inhibition of one of the measured metabolites on one of the irreversible enzymes in the model in an unbiased manner. Since we assumed the reversible reactions to be close to equilibrium, we lumped glucose 6-phosphate with fructose 6-phosphate and 2/3-phosphoglycerate with PEP, leading to 120 possible regulatory interactions between enzymes and metabolites. Combining those 120 interactions in pairs resulted in a set of 6480 pairwise combination models. To assess the ability of the pairwise models to describe the experimental data, we used the Akaike Information Criterion that penalizes fits obtained with a more complex model^34^ and compared this to the value of the base model to evaluate whether the implementation of additional allosteric regulation improved the simulated concentrations compared to the measured data. About 21% (1383/6480) of the pairwise models improved the fit with respect to the base model, indicating that indeed allosteric regulation is required.

**Figure 5:**
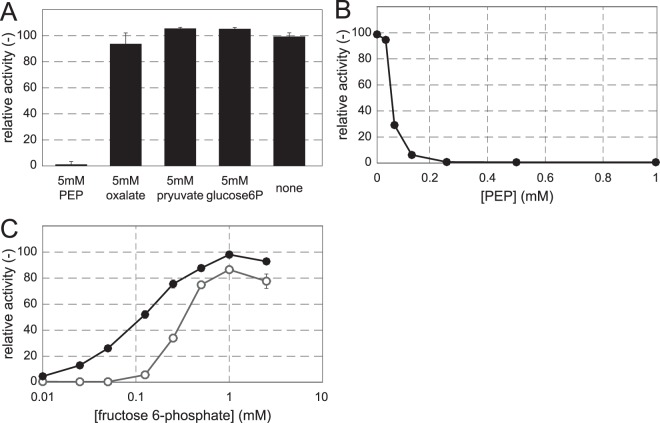
*In vitro* verification of allosteric inhibition by phosphoenolpyruvate (PEP) on phosphofructokinase (PfkA). (**A**) Relative activity of PfkA with 0.125 mM fructose 6-phosphate and 5 mM of predicted regulators. (**B**) PfkA activity at different PEP concentrations in the presence of 0.125 mM fructose 6-phosphate normalized to the maximum activity observed. (**C**) PfkA activity without effector (black solid line with filled circles), and with 0.1 mM PEP (grey solid like with open circles) normalized to the maximum activity observed.

To generate specific hypotheses on the most probably allosteric mechanisms, we ranked the interactions based on their frequency of occurrence in improved models and the Akaike Information Criterion of the best pairwise model for each interaction. Only eight interactions improved every model in which they occurred (Table 2). Strikingly all of those interactions involved regulation of Pfk or fructose-1,6-biphosphatase (FBPase). The top-ranking interactions included the only allosteric interaction for *B. subtilis* reported in the BRENDA database; i.e. activation of FBPase by PEP^35^. To confirm allosteric regulation of Pfk, we performed *in vitro* assays with the purified enzyme of wild-type *B. subtilis* and found that PEP indeed inhibits Pfk with K_i_ = 0.04 mM (Fig. 5), as had been described for *B. licheniformis*^36^. While inhibition of Pfk by pyruvate and activation of Pfk by glucose 6-phosphate or fructose 6-phosphate would be plausible mechanisms because models containing these interactions scored high, *in vitro* activity of these enzymes was insensitive to the presence of these metabolites (Fig. 5). Finally, we demonstrated that PEP inhibition of Pyk and activation of FBPase was sufficient to explain the dynamic metabolome response (Fig. 4B), in particular the FBP dynamics. Thus we provide evidence that reversal of glycolytic flux in *B. subtilis* is primarily regulated by the transcription factors GlcT and CggR in combination with antagonistic allosteric regulation of the Pfk/FBPase pair in upper glycolysis.

**Table 2:** Best interactions found in models with pairwise interaction combinations. PYR = pyruvate, OAA = oxaloacetate, PEP = phosphoenolpyruvate, HexP = hexose phosphate (G6P or F6P), Pfk = phosphofructokinase, Fbp = fructose 1,6-biphosphatase.

8 best interactions
OAA(−)Pfk
HexP(+)Pfk
PEP(−)Pfk
PYR(−)Pfk
HexP(−)Fbp
PYR(+)Fbp
PEP(+)Fbp
OAA(+)Fbp

## Discussion

In sharp contrast to the extensive knowledge on gene regulation^19,37–41^, we have much less knowledge of metabolic regulation in *B. subtilis* than in *E. coli*. Different regulation mechanisms in glycolysis lead to very different dynamics of glucose metabolism. During gluconeogenic metabolism *E. coli* maintains the full capacity for instantaneous glucose catabolism because the glycolytic enzymes are still expressed and allosteric regulation enables a very rapid flux reversal, as was described before^13^. In contrast, in *B. subtilis* transcriptional regulation by GlcT and CggR represses genes encoding for glycolytic enzymes, which prevents instantaneous uptake and metabolism of glucose during gluconeogenic growth. Through our combined quantitative metabolomics and kinetic modeling approach, we generated hypotheses for the *in vivo* relevant allosteric regulations beyond the initial transcriptional response of *B. subtilis* upon exposure to glucose. We addressed the uncertainties of the approach by sampling the free parameters in a physiological range and subsequently conducting *in vitro* enzyme assays to reveal which of the predicted interactions are true. Despite intrinsic limitations of this type of mechanistic modeling of biological systems, we identified the most likely point of regulation. Since the Pfk and FBPase enzyme pair in upper glycolysis is similarly transcribed in glycolytic and gluconeogenic conditions^19,41,42^, additional allosteric regulation is needed to prevent futile cycling. We demonstrate that allosteric activation of FBPase^35^ and the newly identified inhibition of Pfk by the lower glycolysis intermediate PEP are the key regulation mechanisms that presumably prevent futile cycling.

As can be expected for organisms featuring catabolite repression^9^, *E. coli* and *B. subtilis* are both incapable of thriving immediately on a gluconeogenic carbon source when grown on glucose. For *B. subtilis* this is irrespective of whether the gluconeogenic carbon source is non-preferred or the preferred malate. Even though there is immediate uptake and metabolism of the gluconeogenic substrates, as was shown before for co-metabolism^14^, flux reversal into upper glycolysis does not occur, and hence growth cannot commence immediately. The reason is presumably a limitation in substrate uptake because isotopic tracer label propagates only slowly into the TCA cycle.

The striking difference between *E. coli* and *B. subtilis* in response to glucose shows that not only carbon catabolite repression, but also the transcriptional activation of the genes required to make use of the preferred carbon source dictates an organism’s ability to adapt to its environment. The question remains why *B. subtilis* does not express the PTS and *eno*-operon constitutively to allow instantaneous use of glucose. Notably, the natural habitat of *B. subtilis* is in the soil, where the environmental conditions are changing at slower time scales than in the gut-habitat where *E. coli* thrives. The additional protein cost for constitutive expression of the enzymes necessary for instantaneous glucose use, might not outweigh the benefit of potentially fast adaptation^14^.

## Electronic supplementary material

Supplementary information
