# Supplementary material for: Capacity for instantaneous catabolism of preferred and non-preferred carbon sources in Escherichia coli and Bacillus subtilis
Source: Sci Rep. 2018 Aug 6;8:11760. doi: 10.1038/s41598-018-30266-3 (PMC6079084; doi:10.1038/s41598-018-30266-3)
Supplement: Supplementary file 1 — Supplementary information [file 41598_2018_30266_MOESM1_ESM.pdf]

Capacity for instantaneous catabolism of preferred and non-preferred carbon sources in *Escherichia coli* and *Bacillus subtilis*

Marieke F. Buffing[1,2], Hannes Link[3], Dimitris Christodoulou[1,2], and Uwe Sauer[1,*]

[1] Institute of Molecular Systems Biology, ETH Zurich, Zurich, Switzerland

[2] Life Science Zurich PhD Program on Systems Biology, Zurich, Switzerland

[3] Max Planck Institute for Terrestrial Microbiology, Marburg, Germany

* Corresponding author. Tel: +41 44 633 36 72; E-mail: sauer@imsb.biol.ethz.ch

Supplementary information

Supplement – table S1

Table S1 – Growth rate and carbon source uptake rates. C = concentration, μ = growth rate, q_s = uptake rate.

Carbon source	μ h^{-1}	Substrate	C $g\ L^{-1}$	q_s $mmol\ h^{-1}\ gcdw^{-1}$	Reference
Bacillus subtilis **168 trp+**					
glucose	0.59 ± 0.02	glucose	3	7.63 ± 0.11	19
pyruvate	0.17 ± 0.01	pyruvate	5	8.26 ± 0.23	19
malate	0.57 ± 0.05	malate	5	26.51 ± 2.01	19
glucose and malate	0.75 ± 0.01	glucose	2	5.95 ± 1.10	19
		malate	4	14.60 ± 1.56	19
Escherichia coli **BW 25113**					
glucose	0.65 ± 0.01	glucose	3	9.65 ± 0.04	6
pyruvate	0.39 ± 0.01	pyruvate	5	26.71 ± 0.79	6

Supplement – model description

Irreversible reactions

Reaction 1 – glucose specific phosphotransferase system (PTSg)

$$v_{PTSg} = v_{max,PTSg} \frac{c_{glucose}}{c_{glucose} + K_{PTSg,glucose}}$$

Reaction 2 – 6-phosphofructokinase (Pfk)

$$v_{Pfk} = v_{max,Pfk} \frac{c_{F6P}}{c_{F6P} + K_{Pfk,F6P}}$$

Reaction 3 – fructose-1,6-biphosphatase (Fbp)

$$v_{Fbp} = v_{max,Fbp} \frac{c_{FBP}}{c_{FBP} + K_{Fbp,FBP}}$$

Reaction 4 – glucose 6-phosphate dehydrogenase (G6PDH)

$$v_{G6PDH} = v_{max,G6PDH} \frac{c_{G6P}}{c_{G6P} + K_{G6PDH,G6P}}$$

Reaction 5 – phosphoenolpyruvate carboxykinase (Pck)

$$v_{Pck} = v_{max,Pck} \frac{c_{OAA}}{c_{OAA} + K_{Pck,OAA}}$$

Reaction 6 – pyruvate kinase (Pyk)

$$v_{Pyk} = v_{max,Pyk} \frac{c_{PEP}}{c_{PEP} + K_{Pyk,PEP}}$$

Reaction 7 – pyruvate carboxylase (Pyc)

$$v_{Pyc} = v_{max,Pyc} \frac{c_{PYR}}{c_{PYR} + K_{Pyc,PYR}}$$

Reaction 8 – pyruvate uptake

$v_{PYRup} = v_{max,PYRup}$ if $c_{PYR,extern} \neq 0$

$v_{PYRup} = 0$ if $c_{PYR,extern} \neq 0$

Reaction 9 – pyruvate dehydrogenase (Pdh)

$$v_{Pdh} = v_{max,Pdh} \frac{c_{PYR}}{c_{PYR} + K_{Pdh,PYR}}$$

Reaction 10 – glyceraldehyde-3-phosphate dehydrogenase A (GapA)

$$v_{GapA} = v_{max,GapA} \frac{c_{DHAP}}{c_{DHAP} + K_{GapA,DHAP}}$$

Reaction 11 – glyceraldehyde-3-phosphate dehydrogenase B (GapB)

$$v_{GapB} = v_{max,GapB} \frac{c_{xPG}}{c_{xPG} + K_{GapB,xPG}}$$

Reversible reactions

Reaction 12/13 – glucose 6-phosphate isomerase (Pgi)

$$v_{Pgi}^{+} = k_{Pgi}^{+} \times c_{G6P}$$

$$v_{Pgi}^{-} = k_{Pgi}^{-} \times c_{F6P}$$

Reaction 14/15 – fructose-1,6-biphosphate aldolase (Ald)
GAP and DHAP are assumed to be in equilibrium by triose phosphate isomerase

$$v_{Ald}^{+} = k_{Ald}^{+} \times c_{FBP}$$

$$v_{Ald}^{-} = k_{Ald}^{-} \times c_{DHAP} \times c_{DHAP}$$

Reaction 16/17 – enolase (Eno)

$$v_{Eno}^{+} = k_{Eno}^{+} \times c_{xPG}$$

$$v_{Eno}^{-} = k_{Eno}^{-} \times c_{PEP}$$

Allosteric interactions

$$v_{max,i}^{*} = v_{max,i} \times \prod_{j} (\frac{c_j}{c_{j,0}})^{a_{i,j}}$$

In which v = kinetic rate, c = concentration, a = allosteric interaction, i = enzyme catalyzing reaction, j = effector metabolite. In the base model all values for a are set to zero, making the power law equal to one.

Parameterization

Futile cycling between Pfk and Fbpase

$$\frac{v_{Fpb} - v_{Pfk}}{v_{Fbp}} = 0.5 - 1$$

Futile cycling between GapA and GapB

$$\frac{v_{GapB} - v_{GapA}}{v_{GapB}} = 0 - 1$$

Pyk flux

$$F_{Pyk} = (0 - 0.2) \times F_{PYRup}$$

Pdh flux

$$F_{Pdh} = (0.7 - 0.75) \times F_{PYRup}$$

Pyc flux

$$F_{Pyc} = F_{PYRup} + F_{Pyk} - F_{Pdh}$$

Model ranking

Sum of squared errors

$$SSE = \sum_{m}^{6} \sum_{n}^{6} (\tilde{c}^{*}_{m.n} - \tilde{c}_{m,n})^2$$

With m = 6 metabolite concentrations, n = 6 time points, $\tilde{c}^{*}_{m.n}$ = simulated relative metabolite concentrations, $\tilde{c}_{m,n}$ = measured relative metabolite concentrations.

Akaike information criterion

$$AIC = N \log\left(\frac{SSE}{N}\right) + 2K$$

$$\Delta AIC_{pairwise\ model} = AIC_{base\ model} - AIC_{pairwise\ model}$$

With N = total number of residuals, and K = number of parameters.

The final ranking of an interaction was the sum of the ΔAIC and the frequency with which the interaction improved a model compared with respect to the base model.
